# The 2024–2025 Pertussis outbreak in children in Chengdu: A large-scale retrospective cross-sectional study

**DOI:** 10.1371/journal.pone.0351127

**Published:** 2026-07-22

**Authors:** Xu Zhang, Hongjia Chen

**Affiliations:** Chengdu Women's and Children's Central Hospital, School of Medicine, University of Electronic Science and Technology of China, Chengdu, China; Universidad Nacional de la Plata, ARGENTINA

## Abstract

**Objective:**

To characterize the epidemiological features of pertussis among pediatric patients tested by polymerase chain reaction (PCR) at a tertiary children's hospital in Chengdu from May 2024 to April 2025, with particular emphasis on temporal variation and age distribution, to inform regional prevention strategies.

**Methods:**

This retrospective cross-sectional study included 19,084 pediatric patients who underwent PCR testing for *Bordetella pertussis*. Multivariable logistic regression and subgroup analyses were used to examine associations between PCR positivity and season, age group, sex, and visit type.

**Results:**

The overall PCR positivity rate was 20.47% (3,905/19,084). Summer had the highest positivity rate (25.62%) and accounted for the majority of positive cases (67.12%, 2,621/3,905), followed by spring (21.50%; 23.58% of cases, 921/3,905). The monthly number of positive cases peaked in June 2024 (n = 1,078). School-aged children (6 to <12 years) represented the largest proportion of positive cases (56.72%, 2,215/3,905) and had significantly higher PCR positivity than other age groups (χ² = 1098.21, df = 4, *P* < 0.001). In multivariable analysis, school-aged children had higher odds of PCR positivity than adolescents (adjusted OR = 1.55, 95% CI 1.23–1.95). No significant association with sex was observed. Outpatients showed higher PCR positivity than inpatients (21.41% vs. 13.66%; crude OR = 1.72, 95% CI 1.52–1.95, *P* < 0.001). The outpatient advantage was most pronounced in spring (crude OR = 1.95) and summer (crude OR = 1.44), with a significant interaction by season (*P* = 0.013).

**Conclusion:**

This hospital-based study identified a substantial burden of PCR-confirmed pertussis among symptomatic children in Chengdu during the study period, with a prominent peak from May to July 2024 and the highest positivity among school-aged children. These findings may partly reflect waning vaccine-induced immunity and increased social contact in school settings, and support strengthened surveillance, timely PCR testing, and targeted pertussis prevention strategies for school-aged children.

## Background

Pertussis, a highly contagious respiratory tract infection caused by *Bordetella pertussis*, continues to pose a major global public health challenge. Despite decades of widespread vaccination, many countries have experienced concerning resurgences of the disease over the past decade [1]. In China, the routine pertussis vaccination program introduced in the 1980s initially brought the disease under effective control. However, since the 2010s, reported cases have increased sharply, with an unprecedented surge following the COVID-19 pandemic. By August 2024, more than 450,000 cases and 24 deaths had been reported nationwide, indicating the re-emergence of this public health threat [2].

The COVID-19 pandemic and its associated non-pharmaceutical interventions (NPIs)—including mask wearing, social distancing, school closures, and reduced healthcare-seeking behavior—substantially reduced population exposure to B. pertussis [3]. This prolonged period of low pathogen circulation led to an “immunity debt”—a growing pool of susceptible individuals who lacked both infection-induced immunity and recent vaccine-boosted protection [3]. Following the relaxation of these measures in late 2022 and early 2023, China, like many other countries, witnessed a sharp rebound in pertussis incidence [2], a trend also observed in other countries such as Italy [4]. This phenomenon underscores the urgent need for updated epidemiological data to inform vaccination policy and public health preparedness [2].

The switch from whole-cell to acellular pertussis vaccines has important immunological consequences. Although acellular vaccines have an excellent safety profile, they elicit a Th2-biased immune response that wanes more rapidly than the Th1/Th17 response induced by whole-cell vaccines or natural infection [5]. Protection against pertussis declines significantly within 3–5 years after the last acellular dose [6]. Consequently, susceptibility accumulates in school-aged children. Nationwide data from China show that the proportion of pertussis-positive cases among school-aged children (6–16 years) rose markedly from 2.0% in 2018 to 40.8% in 2024 [7]. A study in Zhejiang Province further revealed that 68.4% of fully vaccinated cases occurred after 6 years of age, with a median interval of 61.45 months between the last vaccine dose and disease onset [8]. Together, these findings indicate that the observed age shift toward older children in the current epidemic is closely linked to waning vaccine-induced immunity [7,8].

In addition, the ongoing pertussis epidemic in China exhibits distinct molecular epidemiological features. In recent years, macrolide-resistant B. pertussis MT28 clones carrying the ptxP3 promoter mutation have spread rapidly nationwide [9]. In Shanghai, the prevalence of ptxP3-MT28 isolates increased from 0% before 2020 to over 98.2% in 2022, accompanied by a rise in macrolide resistance from 36.4% to 97.2% [9,10]. The emergence of these resistant strains has not only complicated clinical management but may also contribute to age shifts through vaccine escape mechanisms [10]. Understanding this molecular background is essential for a comprehensive appreciation of the complexity underlying the current pertussis resurgence.

Accurate and timely diagnosis is critical for pertussis surveillance and control. Traditional methods—bacterial culture and serology—have notable limitations. Culture sensitivity is low, especially after antibiotic use, and requires specialized media with prolonged incubation. Serology suffers from poor specificity and lacks standardized cut-offs for recent infection [11]. In contrast, real-time quantitative PCR offers high sensitivity and specificity, rapid turnaround, and the ability to detect B. pertussis even in mild or atypical cases. Recent studies have demonstrated that multiple real-time PCR assays achieve positive percent agreement (PPA) of up to 98.4% for B. pertussis, confirming their robust diagnostic performance [12]. These characteristics make PCR particularly suitable for large-scale epidemiological investigations, enabling the capture of cases that might be missed by conventional approaches.

Chengdu, the largest city in Southwest China with a population of approximately 20 million, has experienced notable shifts in pediatric respiratory disease patterns in the post-pandemic era. However, large-scale PCR-based epidemiological studies of pertussis are lacking in this region. Given the pressing need for region-specific evidence, this retrospective cross-sectional study aimed to characterize the epidemiological features of pertussis among children in Chengdu from May 2024 to April 2025, with particular emphasis on seasonal variation and age distribution, to guide targeted vaccination strategies and public health preparedness.

## Methods

### Study design and subjects

This retrospective cross-sectional study was approved by the Ethics Committee of Chengdu Women's and Children's Central Hospital (Approval No.: 2025〔77〕, dated 17/06/2025). The committee waived the requirement for written informed consent due to the retrospective and anonymized nature of the data. All data were accessed and analyzed on 18/06/2025.

The enrolled population consisted of children with respiratory symptoms, particularly paroxysmal or spasmodic cough, characteristic of pertussis. Patients older than 18 years or those with incomplete clinical data were excluded.

### Variable grouping

Age groups were defined a priori to align with national diphtheria, tetanus, and acellular pertussis (DTaP) vaccination milestones and expected immunity gaps: Infant (0 to <1 year), Toddler (1 to <3 years), Preschooler (3 to <6 years), School-aged children (6 to <12 years), and Adolescent (12 to <18 years).

Seasons were defined as follows: Spring (March–May), Summer (June–August), Autumn (September–November), and Winter (December–February). Due to the study period spanning from May 2024 to April 2025, May 2024 was categorized as Spring, and March–April 2025 were also included in Spring.

### Specimen collection and detection methods

Trained healthcare personnel collected throat swab specimens using sterile polyester swabs. The swabs were transported to the laboratory at 2–8°C. All samples were stored at 2–8°C and tested within 24 hours of collection. Nucleic acids were extracted using the NATCH 48 automated nucleic acid extraction system. PCR amplification and detection were performed using the Gentier 96E real-time PCR system and the *Bordetella pertussis* Nucleic Acid Detection Kit (fluorescence probe method; Yilifang Biotech, China; NMPA Registration No. 20203400152).

The kit targets a highly conserved region of the B. pertussis genomic DNA using specific primers and a fluorescent probe. B. pertussis signals were monitored in the FAM channel, while internal control (IC) signals (recombinant plasmid) were monitored in the VIC/HEX channel to monitor PCR reaction validity. Results were interpreted as follows:

Positive: S-shaped amplification curve in FAM channel with Ct value ≤ 38.

Negative: No amplification curve in FAM channel (Ct > 38) and IC Ct value ≤ 34 in VIC/HEX channel.

Invalid: No amplification curve or Ct > 34 in VIC/HEX channel (sample retested).

All procedures strictly followed the manufacturer's protocol. Laboratory personnel received standardized training to ensure accuracy and prevent cross-contamination.

According to the manufacturer's performance data, the kit demonstrated a positive percent agreement of 94.4% and a negative percent agreement of 100% compared to sequencing, with a total agreement of 97.5%. The limit of detection (LoD) was 1.0 × 10⁴ copies/mL.

Cross-reactivity testing performed by the manufacturer showed no reaction with the following 23 respiratory pathogens: Bordetella parapertussis, Bordetella bronchiseptica, Bordetella holmesii, influenza A virus, influenza B virus, respiratory syncytial virus (RSV), adenovirus, parainfluenza virus type 1, parainfluenza virus type 2, parainfluenza virus type 3, Epstein-Barr virus (EBV), rhinovirus, bocavirus, coronavirus NL63, coronavirus OC43, Mycoplasma pneumoniae, Chlamydia pneumoniae, Legionella pneumophila, Coxiella burnetii, Streptococcus pneumoniae, Klebsiella pneumoniae, Haemophilus influenzae, and Staphylococcus aureus.

### Statistical analysis

Statistical analyses were performed using R software (version 4.4.3), and GraphPad Prism (version 10.1.2) was used for data visualization. Categorical variables, such as season, age group, gender, and visit type, were summarized as frequencies and percentages. The Chi-square test was used to assess associations between these variables and pertussis positivity. Binary logistic regression identified independent predictors of pertussis positivity, with results reported as odds ratios (ORs) and 95% confidence intervals (CIs). Subgroup analyses explored the association between visit type and PCR positivity across different seasons, age groups, and genders, and tested for interaction effects. Temporal trends from May 2024 to April 2025 were analyzed to assess seasonal fluctuations. All statistical tests were two-sided, and a *P*-value < 0.05 was considered statistically significant.

## Results

**Analysis of the impact of season, age, gender, and visit type on pertussis positivity rates**

Among 19,084 pediatric patients tested for pertussis by PCR (May 2024–April 2025), 3,905 were positive (20.47% positivity). The demographic and clinical characteristics of the cohort are summarized in Table 1. Seasonal distribution of positive cases showed summer had the highest case burden (n = 2,621, 67.12% of total positives), followed by spring (n = 921, 23.58%). Summer also had the highest positivity rate (25.62%), followed by spring (21.50%), while autumn and winter had much lower positivity rates (8.62% and 5.87%, respectively). School-aged children comprised most cases (56.72%, n = 2,215) with peak positivity (33.39%). Gender differences were non-significant (*P* = 0.103). Outpatients accounted for 91.83% of cases (n = 3,586) with higher positivity than inpatients (21.41% vs 13.66%, *P* < 0.001).

**Table 1:** Distribution of pertussis PCR positivity by season, age group, gender, and visit type.

Variables	Total (n = 19084)	Negative (n = 15179)	Positive (n = 3905)	Statistic	*P*
Season, n(%)				χ²=484.23	< 0.001
Spring	4283 (22.44)	3362 (78.50)	921 (21.50)		
Summer	10230 (53.61)	7609 (74.38)	2621 (25.62)		
Autumn	3446 (18.06)	3149 (91.38)	297 (8.62)		
Winter	1125 (5.89)	1059 (94.13)	66 (5.87)		
Age, n(%)				χ²=1098.21	< 0.001
Infant	1682 (8.81)	1426 (84.78)	256 (15.22)		
Toddler	2513 (13.17)	2267 (90.21)	246 (9.79)		
Preschooler	7820 (40.98)	6736 (86.14)	1084 (13.86)		
School-aged child	6633 (34.76)	4418 (66.61)	2215 (33.39)		
Adolescent	436 (2.28)	332 (76.15)	104 (23.85)		
Gender, n(%)				χ²=2.66	0.103
Female	9049 (47.42)	7152 (79.04)	1897 (20.96)		
Male	10035 (52.58)	8027 (79.99)	2008 (20.01)		
Visit type, n(%)				χ²=75.60	< 0.001
Inpatient	2335 (12.24)	2016 (86.34)	319 (13.66)		
Outpatient	16749 (87.76)	13163 (78.59)	3586 (21.41)		

χ²: Chi-square test

### Distribution of Pertussis-Positive Cases by Age, Gender, and Patient Type

**Fig 1:**
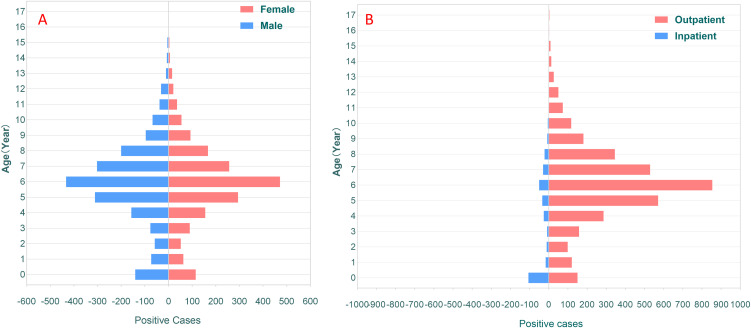
Age-specific distribution of pertussis-positive cases by gender and patient type (bidirectional bar chart). Note that the bars represent raw case counts, reflecting clinical burden rather than population incidence rates.

Fig 1A and 1B present bidirectional bar charts illustrating the age-specific distribution of pertussis-positive pediatric cases by gender and patient type. In the gender-based distribution, the number of cases peaked between ages 5 and 7 for both males and females, with the highest numbers observed at age 6 (433 males, 471 females). Overall, the gender distribution was balanced across age groups, with no apparent disparity observed. In the patient type distribution, most positive cases were recorded in outpatient settings. The number of outpatient cases consistently exceeded inpatient cases across all age groups, with the largest difference at age 6 (854 outpatients vs. 50 inpatients). Inpatient cases were rare, particularly after age 10, with almost no hospitalized cases recorded in adolescents.

### Temporal and age-specific distribution of pertussis-positive cases

**Fig 2:**
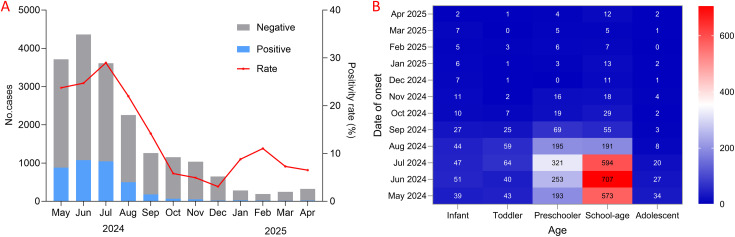
Monthly distribution of pertussis PCR-positive cases and positivity rates from May 2024 to April 2025, stratified by age group. A: bar chart (case counts) and line chart (positivity rate, right y-axis). B: heatmap of raw positive case counts by age group; darker color indicates more cases.

The number of positive pertussis cases and the positivity rate exhibited significant seasonal variation, as shown in the line chart in Fig 2. In May 2024, there were 882 positive cases (23.75% positivity rate). The number of positive cases increased to a peak of 1,078 cases in June 2024, followed by 1,046 cases in July 2024. Thereafter, cases declined sharply to a low of 20 in December 2024 (3.07% positivity rate). In early 2025, cases rose slightly. Overall, positive cases were more frequent in summer (June–August) and fewer in winter (November–February), indicating seasonal fluctuation. The heatmap illustrates a peak in positive cases from May to July—especially in June and July—with a notable increase among school-aged children. Toddlers and preschoolers also exhibited increased cases during this period. Cases were generally lower during other months—particularly in winter—with fewer cases among infants and adolescents.

**Logistic regression analysis results: the impact of season, age group, and visit type on positive cases**

**Fig 3:**
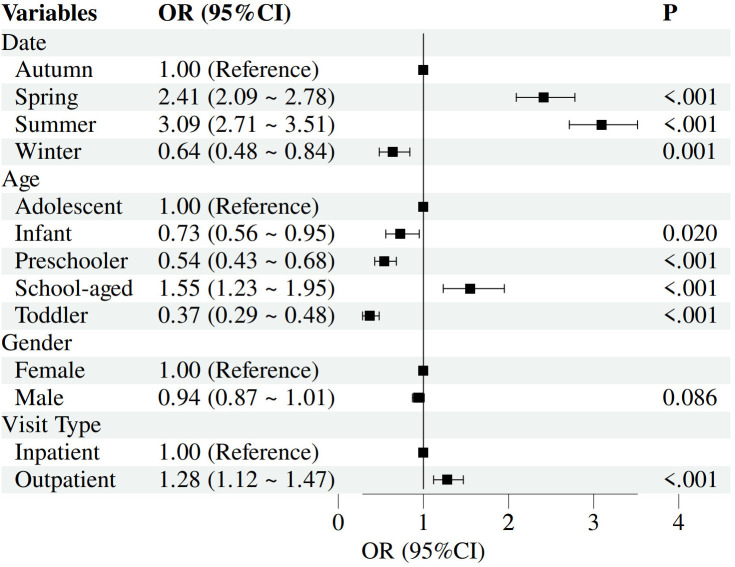
Multivariable logistic regression analysis of factors associated with pertussis PCR positivity. Reference groups: Season – Autumn; Age – Adolescents; Gender – Female; Visit Type – Inpatient. Error bars represent 95% confidence intervals.

Multivariable logistic regression (Fig 3) showed that compared to autumn, summer (aOR = 3.09, 95% CI 2.71–3.51) and spring (aOR = 2.41, 95% CI 2.09–2.78) were associated with significantly higher odds of PCR positivity, while winter was associated with lower odds (aOR = 0.64, 95% CI 0.48–0.84) (all *P* < 0.001). For age, school-aged children had higher odds than adolescents (aOR = 1.55, 95% CI 1.23–1.95, *P* < 0.001), whereas infants (aOR = 0.73, 95% CI 0.56–0.95, *P* = 0.020), toddlers (aOR = 0.37, 95% CI 0.29–0.48, *P* < 0.001), and preschoolers (aOR = 0.54, 95% CI 0.43–0.68, *P* < 0.001) had lower odds. Sex was not significantly associated (aOR = 0.94, 95% CI 0.87–1.01, *P* = 0.086). Outpatients had higher odds than inpatients (aOR = 1.28, 95% CI 1.12–1.47, *P* < 0.001).

### Subgroup analysis of PCR positivity odds by visit type

**Fig 4:**
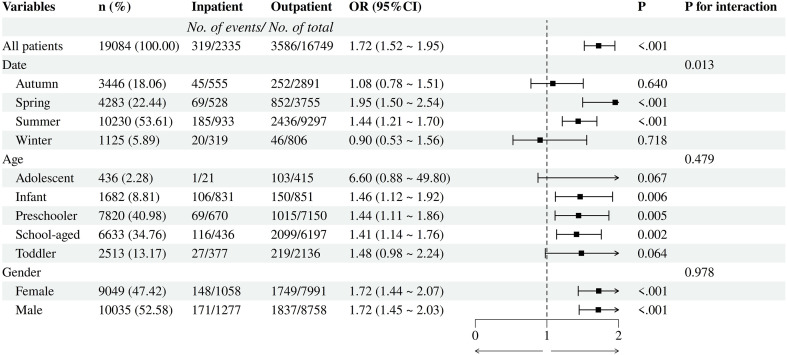
Subgroup analysis of PCR positivity odds: Outpatients vs. Inpatients.

Subgroup analysis (Fig 4) showed that overall, outpatients had significantly higher odds of testing positive than inpatients (crude OR = 1.72, 95% CI 1.52–1.95, *P* < 0.001). The outpatient advantage varied significantly by season (interaction *P* = 0.013), being strongest in spring (crude OR = 1.95, 95% CI 1.50–2.54) and summer (crude OR = 1.44, 95% CI 1.21–1.70), but absent in autumn (crude OR = 1.08, 95% CI 0.78–1.51, *P* = 0.640) and winter (crude OR = 0.90, 95% CI 0.53–1.56, *P* = 0.718). This advantage was consistent across age groups (interaction *P* = 0.479), with crude ORs ranging from 1.41 (95% CI 1.14–1.76) in school-aged children to 1.48 (95% CI 0.98–2.24) in toddlers, and was nearly identical in females (crude OR = 1.72, 95% CI 1.44–2.07) and males (crude OR = 1.72, 95% CI 1.45–2.03) (interaction *P* = 0.978). Adolescents showed a wide confidence interval (crude OR = 6.60, 95% CI 0.88–49.80) due to the small number of hospitalized cases (n = 21).

## Discussion

To the best of our knowledge, this is the first large-scale, hospital-based epidemiological study of pertussis in southwestern China using PCR as the sole diagnostic method. In this retrospective cross-sectional study, we analyzed PCR test results from 19,084 children who presented to a tertiary pediatric specialty hospital in Chengdu between May 2024 and April 2025, identifying 3,905 laboratory-confirmed cases (overall positivity rate 20.47%). It should be noted that this positivity rate reflects the detection rate among symptomatic children seeking care at a referral hospital and does not represent the true prevalence in the general pediatric population of Chengdu. This study fills a critical gap in pertussis epidemiological data for southwestern China. The COVID-19 pandemic and associated NPIs, such as school closures and masking, substantially reduced population exposure to B. pertussis, leading to an ‘immunity debt’ that likely contributed to the scale of the 2024–2025 outbreak [3,13].

The outbreak exhibited a marked seasonal pattern: summer (June–August) accounted for 67.12% (n = 2,621) of all positive cases, with the highest positivity rate (25.62%). Case numbers peaked in June 2024 (n = 1,078), remained elevated in July (n = 1,046), and declined substantially in August (n = 497). This summer-dominant pattern is consistent with post-COVID-19 pertussis epidemics observed across China and with seasonal variation reported in Northern Vietnam [14]. A recent multicenter study confirmed that the 2024 national outbreak peak in China occurred between April and July, closely mirroring our observations [13]. *Bordetella pertussis* grows optimally at 35–37°C, and Chengdu's hot, humid summer climate favors pathogen survival. In addition, during hot weather, children spend more time indoors in poorly ventilated air-conditioned spaces, with reduced outdoor activity and increased indoor crowding, further promoting droplet transmission. This re-emerging threat despite widespread immunization has been highlighted in recent analyses [5]. These high-temperature and high-humidity conditions, rather than the calendar months per se, are presumed risk factors for increased transmission.

School-aged children (6–12 years) bore the highest disease burden, comprising 56.72% (n = 2,215) of all positive cases and showing the highest positivity rate (33.39%). Multivariable logistic regression confirmed that this age group was an independent risk factor compared with adolescents (adjusted odds ratio [aOR] 1.55, 95% CI 1.23–1.95, *P* < 0.001). By contrast, toddlers (1–3 years) had the lowest positivity rate (9.79%), likely attributable to recent completion of primary immunization. Positivity rates were 15.22% in infants (0–1 year), 13.86% in preschool children (3–6 years), and 23.85% in adolescents (12–18 years). This clear age shift aligns closely with the well-documented rapid waning of acellular pertussis vaccine-induced immunity within 3–5 years after the final dose, as confirmed by a systematic review and meta-analysis [6]. A multicenter study in Hubei involving 20,727 children similarly identified that 5–9-year-olds accounted for 58.2% of PCR-confirmed cases [15]. Collectively, these findings—consistent with recent evidence from Chongqing [16]—underscore the reality of the immunity gap among school-aged children, thereby reinforcing the necessity of China's recent immunization schedule adjustment, which introduced a fifth DTaP dose at age 6 [17].

Outpatients accounted for 91.83% of positive cases, and their PCR positivity rate was significantly higher than that of inpatients (21.41% vs. 13.66%, crude OR = 1.72, 95% CI 1.52–1.95, *P* < 0.001; reference group: inpatients). Subgroup analysis showed that the outpatient positivity advantage was most pronounced in spring (crude OR = 1.95) and summer (crude OR = 1.44, interaction *P* = 0.013). This pattern may reflect the fact that spring corresponds to the early phase of the outbreak, when symptoms are milder and children are more likely to present first to outpatient clinics; in summer, although overall incidence peaks, some severe cases progress rapidly and are admitted directly, thereby attenuating the outpatient advantage. U.S. CDC guidelines state that PCR sensitivity is highest during the catarrhal phase and early paroxysmal phase (within 3–4 weeks of cough onset), when most affected children remain in outpatient care [18]. The Hubei multicenter study also reported that the median age of inpatients (0 years) was significantly younger than that of outpatients (6 years), indicating that younger and more severely ill children are more likely to require hospitalization [15]. These findings underscore that routine PCR screening in outpatient settings during peak seasons can facilitate early diagnosis, reduce misdiagnosis, and effectively interrupt community transmission, conferring important public health value.

Although macrolide-resistant *Bordetella pertussis* MT28 clones have been confirmed to drive the national outbreak [10], this study did not perform molecular typing and therefore cannot evaluate the contribution of specific epidemic strains to the Chengdu outbreak. Recent molecular surveillance in northern China further demonstrated that ptxP3 high-virulence strains surged from 13.5% in 2019–2021 to 93.0% in 2022–2023, with concurrent erythromycin resistance reaching 100% in ptxP3 isolates, and MT28 strains accounted for 66.0% of tested isolates [19]. These findings underscore the rapid clonal expansion of the ptxP3-MT28 lineage as a major driver of the nationwide pertussis resurgence. Future surveillance in southwestern China should incorporate genotypic characterization to monitor the local spread of these epidemic clones.

## Limitations

First, regarding study design and generalizability: This was a single-center, hospital-based retrospective cross-sectional study conducted at a tertiary pediatric hospital in Chengdu. The enrolled population consisted of symptomatic children who underwent PCR testing based on clinical suspicion of pertussis, which may have introduced selection bias and limits the generalizability of the findings to primary care settings, asymptomatic populations, or other regions. Moreover, due to the cross-sectional design, causal relationships cannot be inferred.

Second, regarding data collection and PCR sensitivity: Data on antibiotic use prior to specimen collection and cough duration at the time of sampling were not systematically recorded, both of which may affect PCR sensitivity. Furthermore, the use of throat swabs rather than nasopharyngeal specimens may have led to an underestimation of PCR positivity.

Third, regarding age grouping and missing information: Although age groups were defined a priori to align with DTaP vaccination milestones, grouping children aged 1 to <3 years into a single “toddler” category precluded evaluation of the independent impact of the 18‑month DTaP booster dose, as this group contained both pre‑booster (12–17 months) and post‑booster (18–35 months) children. Additionally, individual-level vaccination histories were unavailable, preventing assessment of vaccine effectiveness and waning immunity. Molecular characterization of the pathogen was also not performed; therefore, this study could not determine the contribution of specific circulating strains (e.g., macrolide-resistant clones) to the local resurgence of pertussis.

Fourth, regarding temporal coverage: Routine pertussis PCR testing at our institution commenced only in May 2024, so cases occurring before this date could not be captured. Moreover, the 12‑month observation period was insufficient to evaluate long‑term epidemiological trends following the implementation of China's revised five‑dose DTaP vaccination schedule in January 2025.

## Conclusions

This study confirms a significant pertussis outbreak among children in Chengdu from 2024 to 2025, characterized by a prominent summer peak and the greatest disease burden in school-aged children. The core findings deliver timely, reliable southwestern data that are consistent with the rationale for national booster immunization strategies and regional precision prevention and control efforts. Multicenter studies incorporating molecular features are warranted to further inform precise pertussis prevention and control in southwestern China.

## Abbreviations

PCR: Polymerase Chain Reaction
DTaP: Diphtheria, Tetanus, and acellular Pertussis vaccine
NPIs: non-pharmaceutical interventions
OR: Odds Ratio
CI: Confidence Interval
aOR: adjusted Odds Ratio
MT28: Macrolide-resistant Bordetella pertussis clone
FAM: Fluorescein Amidite
VIC/HEX: Reporter dye used in real-time PCR for internal control
Ct: Cycle threshold
B. pertussis: *Bordetella pertussis*
ptxP3: Pertussis toxin promoter type 3
